# Evaluation of Prebiotic Activity of Stellariae Radix Polysaccharides and Its Effects on Gut Microbiota

**DOI:** 10.3390/nu15224843

**Published:** 2023-11-20

**Authors:** Hong Wang, Haishan Li, Zhenkai Li, Lu Feng, Li Peng

**Affiliations:** 1 School of Life Sciences, Ningxia University, Yinchuan 750021, China; wh12100912@163.com (H.W.); lhs985233@163.com (H.L.);; 2 College of Resource and Environment and Life Science, Ningxia Normal University, Guyuan 756000, China; 3 Ningxia Natural Medicine Engineering Technology Research Center, Yinchuan 750021, China

**Keywords:** Stellariae Radix, polysaccharides, prebiotic activity, gut microbiota

## Abstract

This study aims to evaluate the prebiotic potential of polysaccharides derived from Stellariae Radix (SRPs) and explore their influence on the gut microbiota composition in mice. *Lactobacillus acidophilus* and *Bifidobacterium longum* were cultivated in an MRS medium, while their growth kinetics, clumping behavior, sugar utilization, pH variation, growth density, and probiotic index were meticulously monitored. Additionally, the impact of crude Stellariae Radix polysaccharides (CSRP) on the richness and diversity of gut microbiota in mice was assessed via 16S rDNA sequencing. The results demonstrated the remarkable ability of CSRPs to stimulate the proliferation of *Lactobacillus acidophilus* and *Bifidobacterium longum*. Moreover, the oral administration of CSRPs to mice led to a noticeable increase in beneficial bacterial populations and a concurrent decrease in detrimental bacterial populations within the intestinal flora. These findings provided an initial validation of CSRPs as a promising agent in maintaining the equilibrium of gut microbiota in mice, thereby offering a substantial theoretical foundation for developing Stellariae Radix as a prebiotic ingredient in various applications, including food, healthcare products, and animal feed. Furthermore, this study presented novel insights for the exploration and utilization of Stellariae Radix resources.

## 1. Introduction

*Lactobacillus acidophilus* and *Bifidobacterium longum* are representative probiotics found in the intestines, and they offer numerous benefits for human health [1]. Probiotics produce beneficial substances for the intestines, such as short-chain fatty acids (SCFAs), lactic acid, and other acidic metabolites, through prebiotics. These substances play a crucial role in maintaining intestinal health [2]. Prebiotics are food components that selectively ferment and alter the composition and activity of beneficial bacteria in the intestines. They mainly consist of non-digestible oligosaccharides and oligose [3]. Many studies have demonstrated that regulating the gut microbiota can reduce the risk of chronic metabolic diseases, including type 2 diabetes, obesity, and non-alcoholic fatty liver disease [4]. The gut microbiota prevents the infection of external pathogens through direct mechanisms (such as competition for nutrients and niches) and indirect mechanisms (such as enhancing the host’s defense) [5]. Therefore, maintaining and regulating the balance of the intestinal microecology is of great significance in disease prevention and promoting overall host health.

Polysaccharides are complex carbohydrates with various biological activities [6]. Plant polysaccharides are typically not directly or fully absorbed by the intestine. Instead, they are broken down into SCFAs and other beneficial metabolites through interactions with intestinal microbes and are then absorbed by the intestine [7]. In recent years, numerous studies have suggested that non-digestible polysaccharides may serve as potential prebiotics for the gut microbiota, leading to changes in microbial structure and composition [8]. For instance, *Lycium barbarum* polysaccharides have been found to promote the growth of *Lactobacillus acidophilus* and increase the proportion of intestinal probiotics, such as *Akkermansia*, *Lactobacillus acidophilus*, and *Prevotellaceae*, in mice [9]. Similarly, Dendrobium huoshanense polysaccharides have been shown to stimulate the production of cytokines, induce the proliferation and differentiation of immune cells in the small intestine, increase the relative abundance of *Lactobacillus*, *Prevotella*, and *Porphyromonas* in the mouse colon, and reduce the abundance of *Helicobacter* and *Chlorotriazole* [10]. Therefore, further research is needed to understand the role of polysaccharides in the gut microbiota and their impact on overall body health.

The traditional Chinese medicine Stellariae Radix is derived from the dried root of *Stellaria dichotoma* L. *var. lanceolata* Bge. [11]. It is characterized by its cold and sweet properties and is known for its efficacy in treating infantile malnutrition and bone tuberculosis [12]. With the advancement of modern medical research, the anti-inflammatory, anti-allergic, and anti-cancer effects of Stellariae Radix have been continuously discovered, enhancing its medicinal value. The effective components and pharmacological effects of Stellariae Radix, as a traditional Chinese medicine, have been extensively reported [13,14,15]. However, there has been no research conducted on the impact of Stellariae Radix polysaccharides (SRPs) on the intestinal microflora.

In this study, *Lactobacillus acidophilus* and *Bifidobacterium longum* were cultured in an MRS medium to monitor the dynamic changes in the growth curve, clump count, pH, growth density, and probiotic index of these two probiotics. Additionally, the effects of crude Stellariae Radix polysaccharides (CSRPs) on the abundance and diversity of gut microbiota in mice were analyzed using 16S rDNA. These findings provide a theoretical basis for the diversified development of food, health products, feed, and other related fields. Furthermore, they offer new insights for the development and utilization of Stellariae Radix resources.

## 2. Materials and Methods

### 2.1. Materials

Stellariae Radix was purchased from Ningxia, China. *Lactobacillus acidophilus* (CGMCC1.3013) and *Bifidobacterium longum* (CGMCC1.1878) were purchased from the China General Microbiological Culture Collection Center. The bacterial strains were inoculated in a broth (MRS) medium and activated under anaerobic conditions (37 °C, 12 h).

### 2.2. Preparation Process of CSRPs

Stellariae Radix medicinal materials were crushed through a 40-mesh sieve and degreased by supercritical CO_2_ extraction (a pressure of 35 MPa, an extraction temperature of 47 °C, a CO_2_ flow rate of 50 L/h, and extraction for 3 h). Then, ultrasonic-assisted extraction was used after concentration and fractional alcohol precipitation, and CSRPs were obtained by the Sevag deproteinization method [16].

### 2.3. Evaluation of Prebiotic Activity In Vitro

The growth and acid production capacity of *Lactobacillus acidophilus* and *Bifidobacterium longum* were determined to evaluate the prebiotic activity of CSRPs [17]. MRS carbohydrate-free broth (Qingdao Top Biotech Co., Ltd., Qingdao, China) was used as the basal medium. In order to cultivate *Bifidobacterium longum*, 5 g/L cysteine was added to the MRS medium as the reducing agent. The existing literature shows that the two probiotics have an optimal growth concentration of 2.5% (*w*/*v*) inulin; thus, 2.5% (*w*/*v*) Glc (negative control without inulin) and 2.5% (*w*/*v*) inulin are selected as the control groups [9]. Then, 2.5% (*w*/*v*) inulin (MRS-L) and 2.5% (*w*/*v*) Glc with different concentrations of CSRPs (2.5%, 5%, 10%, and 15% (*w/v*)) were added to the MRS broth medium as carbohydrate sources, and MRS-L was used as the control group. The broth medium was inoculated with 5% (*v*/*v*) (1−2 × 10^9^ CFU/mL) activated *Lactobacillus acidophilus* or *Bifidobacterium longum*. And the culture medium was incubated under anaerobic conditions for 48 h (37 °C, 120 rpm), and random sampling was performed every 3 h to determine the OD value of the culture medium. In addition, the logistic model in Origin Pro 2019b software [18] was used, and the iterative algorithm used was the Levernberg–Marquardt optimization algorithm. And the algorithm (expression (1)) was as follows, and the fitting growth curves of two bacteria were obtained.

y = A_2_ + (A_1_ − A_2_)/(1 + (X/X_0_)^p) (1)

In the formula, A_1_, A_2_, X_0_, and p are parameters; A_1_ is the deviation degree between the real curve and the model; A_2_ is the growth maximum predicted by the model; X_0_ is the inflection point time, representing the maximum growth rate at this time; and p is the growth rate coefficient, and the curve has a maximum slope at the crossing point (X_0_, A_2_), indicating the maximum growth rate.

The growth number of two probiotic strains was determined via the plate counting method, expressed as log CFU/mL. The control groups are 2.5% (*w/v*) Glc and 2.5% (*w*/*v*) inulin. The broths were inoculated with 5% (*v*/*v*) (10 × 10^9^ CFU/mL) of stationary-phase *Lactobacillus acidophilus* and *Bifidobacterium longum*. The cultures were then incubated at 37 °C for 24 h with agitation (120 rpm) under anaerobic conditions. After incubation, serial dilutions (10^−1^–10^−8^) were performed using sterile normal saline and plated on an MRS agar at 37 °C for 48 h under anaerobic conditions. The results were recorded as CFU/mL of culture. The acid production ability and growth of the two probiotic strains with pH and OD values were determined, and the probiotic values (PIs) were calculated.

### 2.4. Evaluation of Prebiotic Activity In Vivo

Clean-grade ICR male mice (8 weeks old; weight: 20.0 ± 2.0 g) were purchased from the Experimental Animal Center of Ningxia Medical University (Ningxia, China) (Certificate No. SCXK Ningxia 2020-0001). The animal housing and feeding conditions were as follows: temperature of 20–26 °C, relative humidity of 40–70%, alternating light and dark for 12 h, free drinking water, and feeding with standard blocontrol maintenance feed.

The mice were randomly divided into four groups (6 in each group), respectively, the control group (CK), the low-dose group (L), the middle-dose group (M), and the high-dose group (H), and were allowed to acclimate for 1 week before the experiment. The control group (intragastric administration of normal saline), L group (CSRPs: 1 g/kg), M group (CSRPs: 2 g/kg), and H group (CSRPs: 4 g/kg), underwent an intragastric administration of 0.1 mL/10 g continuously for 14 days. After 24 h from the last administration, the blood was taken from the eyeballs of the mice, and fresh feces were collected.

### 2.5. Composition and Diversity of Gut Microbiota

The 16S rDNA amplicon sequencing technology was used to study microbial composition and structure in the mice’s intestine. Total microbial genomic DNA was extracted from the samples using the E.Z.N.A.^®^ soil DNA kit (Omega Bio-tek, Norcross, GA, USA) according to the manufacturer’s instructions. The quality and concentration of DNA were determined with 1.0% agarose gel electropHoresis and a NanoDrop^®^ ND-2000 spectropHotometer (Thermo Scientific Inc., Waltham, MA, USA). The hypervariable region, V3-V4, of the bacterial 16S rRNA gene was amplified with the primer pairs 338F (5′-ACTCCTACGGGAGGCAGCAG-3′) and 806R (5′-GGACTACHVGGGTWTCTAAT-3′) by an ABI GeneAmp^®^ 9700 PCR thermocycler (ABI, Los Angeles, CA, USA). All samples were amplified in triplicate after mixing PCR products of the same sample, and the PCR product was extracted from a 2% agarose gel and purified using the AxyPrep DNA Gel Extraction Kit (Axygen Biosciences, Union City, CA, USA) according to the manufacturer’s instructions and quantified using a Quantus™ Fluorometer (Promega, Madison, WI, USA). An Illumina Pair-End library was constructed, and the sub-library was expanded to perform pairing. Online measurements on the Illumina MiSeq platform were carried out.

Using UPARSE 7.1, the sequences were clustered into operational taxonomic units (OTUs) with 97% consistency. Bacterial annotation of OTU sequences was carried out, and a bacterial annotation analysis was carried out using the Mothur method and the SSUrRNA database of SILVA132 to obtain taxonomic information and the composition of the bacterial colonies of the samples to be tested. Alpha diversity indices for evaluating gut microbial community richness were calculated. A beta diversity analysis was used to investigate the structural variations in the microbial communities using the UniFrac distance.

### 2.6. Statistical Analysis

Data are presented as the mean ± SD, and the statistical analysis was performed using Origin 2019 software (OriginLab Corporation, Northampton, MA, USA). A one-way analysis of variance (ANOVA) plus the post hoc Duncan’s test (SPSS software, version 22.0) were used to evaluate the statistical significance. The Wilcox rank sum test and Tukey test were used to analyze the differences in bacterial diversity between the groups. A bioinformatic analysis of the gut microbiota was carried out using the Majorbio Cloud platform (https://cloud.majorbio.com, accessed on 30 September 2021).

## 3. Results

### 3.1. Composition of Polysaccharide from Stellariae Radix

The SRP consists of Gal, Glc, Xyl, Fru, Man, and Rha, with molar percentages of 61.86%, 32.51%, 4.77%, 0.39%, 0.28%, and 0.19%, respectively. It has a molecular weight of 31,309 Da, and the sugar ring structure of pyran sugar contains α-configuration glycosidic bonds and β-configuration glycosidic bonds [19].

### 3.2. Evaluation of Prebiotic Activity In Vitro

#### 3.2.1. Fitting of Growth Curve

**Figure 1:**
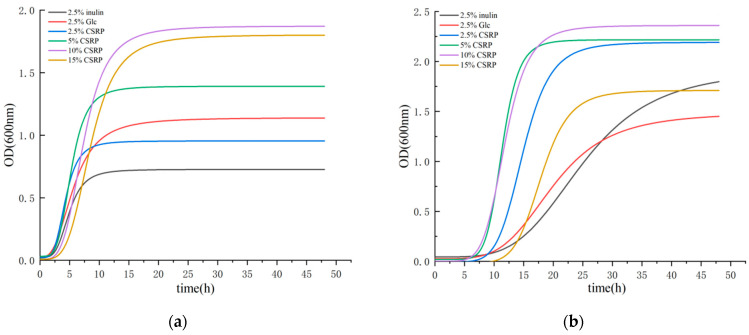
(**a**) Growth fitting curves of *Lactobacillus acidophilus*; (**b**) growth fitting curves of *Bifidobacterium longum*. (The concentration 10% CSRPs is the optimal concentration for *Lactobacillus acidophilus* and *Bifidobacterium longum* growth).

The growth curves of the two probiotic strains are plotted in Figure 1, and the model fitting had determination coefficient (R^2^) values greater than 0.9, indicating a good fitting effect. Figure 1a shows the growth curve of *Lactobacillus acidophilus* with different carbon (2.5% (*w*/*v*) inulin (MRS-L), 2.5% (*w*/*v*) Glc, and different concentrations of CSRP (2.5%, 5%, 10%, and 15% (*w*/*v*))) sources. A lag phase occurred from 0 to 3 h, followed by a log phase from 3 to 12 h. After 12 h, the growth rate slowed down, and from 15 to 48 h, a stationary phase was observed. Figure 1b depicts the growth curve of *Bifidobacterium longum* with different carbon (2.5% (*w*/*v*) inulin (MRS-L), 2.5% (*w*/*v*) Glc, and different concentrations of CSRP (2.5%, 5%, 10%, and 15% (*w*/*v*))) sources. In general, an extended lag phase was observed from 3 to 9 h, followed by a log period from 9 to 18 h. After 18 h, the growth rate decreased, and from 21 to 48 h, a stationary phase was observed.

By referring to Table 1, it can be seen that both probiotic strains exhibited rapid growth in the medium with different carbon (2.5% (*w*/*v*) inulin (MRS-L), 2.5% (*w*/*v*) Glc, and different concentrations of CSRP (2.5%, 5%, 10%, and 15% (*w*/*v*))) sources. In the medium with different carbon sources for *Lactobacillus acidophilus*, the addition of the 2.5% and 5% CSRP groups resulted in a faster entry into the logarithmic growth phase compared to the control group (2.5% inulin and 2.5% Glc) (CSRP group X_0_ < control group X_0_). However, the addition of the 10% and 15% CSRP groups entered the log phase later than the control group, and the former also entered the stationary phase earlier. Similarly, *Bifidobacterium longum* in different carbon-source mediums with different concentrations of CSRP groups entered the log phase faster than the control group (2.5% inulin and 2.5% Glc) (CSRP group X_0_ < control group X_0_). Furthermore, the maximum growth number of both probiotic strains in each CSRP concentration group was greater than the control group (the CSRP group’s *p*-value was greater than that of the control group) after entering the stable phase. These results indicate that the CSRP groups, at each concentration, effectively enhance the metabolic rate of the two beneficial strains and promote their proliferation compared to the control group (2.5% inulin and 2.5% Glc).

#### 3.2.2. Effects of CSRPs on Growth of Lactobacillus Acidophilus and Bifidobacterium Longum

The effects of CSRPs on the growth of *Lactobacillus acidophilus* and *Bifidobacterium longum* were evaluated by measuring the total number of viable bacteria (log10 CFU/mL). According to Table 2, the maximum growth of *Lactobacillus acidophilus* and *Bifidobacterium longum* was observed at a concentration of 10% CSRPs, while the growth showed a downward trend at the highest concentration.

#### 3.2.3. Effects of CSRPs on Acid Production of Two Probiotics

**Figure 2:**
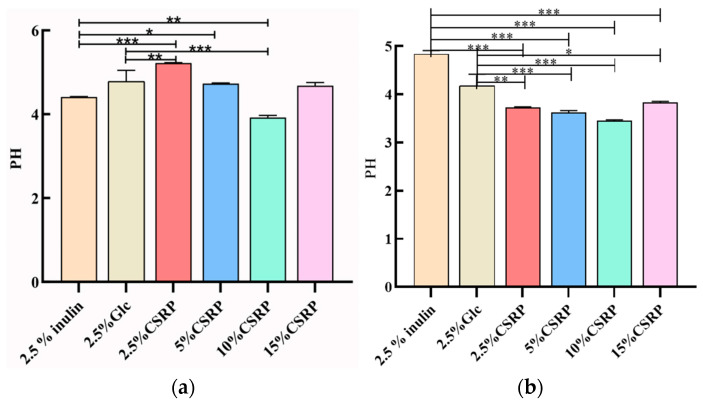
(**a**) Effects of different concentrations of CSRPs on acid production of *Lactobacillus acidophilus*; (**b**) effects of different concentrations of CSRPs on acid production of *Bifidobacterium longum*. (* *p* < 0.05, ** *p* < 0.01, *** *p* < 0.001). (10% CSRPs can promote acid production by probiotics).

Polysaccharides serve as a carbon source that can be decomposed and utilized by probiotics. During this process, the probiotics produce short-chain fatty acids and other substances, leading to a decrease in pH. To evaluate the growth of probiotics, the changes in pH were monitored. In Figure 2, the pH changes of *Lactobacillus acidophilus* (Figure 2a) and *Bifidobacterium longum* (Figure 2b) are shown when CSRPs were used as a carbon source, replacing glucose in the MRS medium.

For Lactobacillus acidophilus, the pH of the 10% CSRP group was significantly lower than that of the control group (2.5% inulin and 2.5% Glc). Similarly, for *Bifidobacterium longum*, the pH of the different concentrations of CSRP groups was significantly lower than that of the control group (2.5% inulin and 2.5% Glc). These results indicate that CSRPs can promote the growth of probiotics by reducing the pH of the medium and consuming the carbon source.

#### 3.2.4. Effects of Different Concentrations of CSRPs on Growth Density of two Probiotic Strains

**Figure 3:**
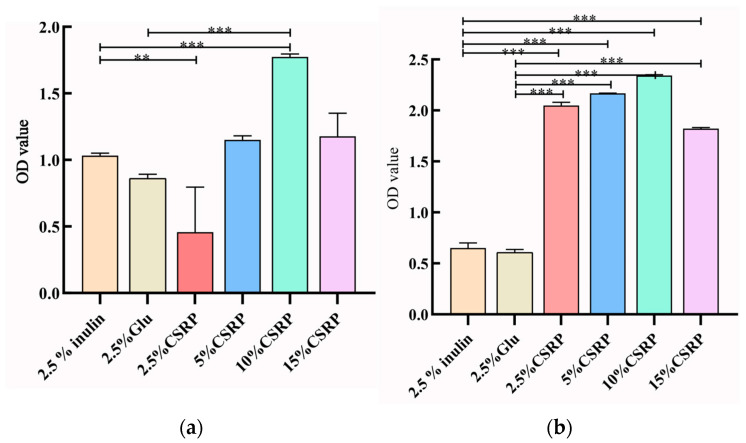
(**a**) Effects of different concentrations of CSRPs on growth density of *Lactobacillus acidophilus*; (**b**) effects of different concentrations of CSRPs on growth density of *Bifidobacterium longum*. ** *p* < 0.01, *** *p* < 0.001). (10% CSRP can promote the growth of probiotics).

The growth of probiotics can also be assessed by measuring the optical density (OD) values of the culture medium, as they reflect the growth rate of the strains. In Figure 3a,b, the OD values of the culture medium for both *Lactobacillus acidophilus* and *Bifidobacterium longum* are recorded. It was observed that with the increase in CSRP concentrations, the OD values gradually increased, reaching their maximum value in the 10% CSRP group. This indicates that higher concentrations of CSRPs promote the growth of probiotics, resulting in higher OD values in the culture medium.

#### 3.2.5. Probiotic Index Calculation of Different Concentrations of CSRPs on Two Probiotic Strains

As shown in the Table 3, the results of the two probiotic strains showed that the addition of 10% CSRPs to the medium had the best effect on promoting the probiotic strains’ growth.

### 3.3. Evaluation of Prebiotic Activity In Vivo

#### Effects of CSRP Intake on the Composition of Cecal Gut Microbiotas

A high-throughput analysis of the 16S rDNA V3-V4 region of the mice feces revealed a total of 1,844,690 sequences and 477 operational taxonomic units (OUTs). The data were stored in NCBI sequence reading files with the login number SRP463499. To assess the richness and diversity of the microbial community, the Chao1 index and Ace index were used to analyze richness, while the Shannon index and Simpson index were used to describe diversity and evenness. Coverage was used to represent the coverage of the microbial community [20]. The results, shown in Table 4, indicated that as the concentration of CSRPs increased, the index values initially increased and then decreased. Among the groups, the M group exhibited the highest richness, while the H group had the highest diversity.

**Figure 4:**
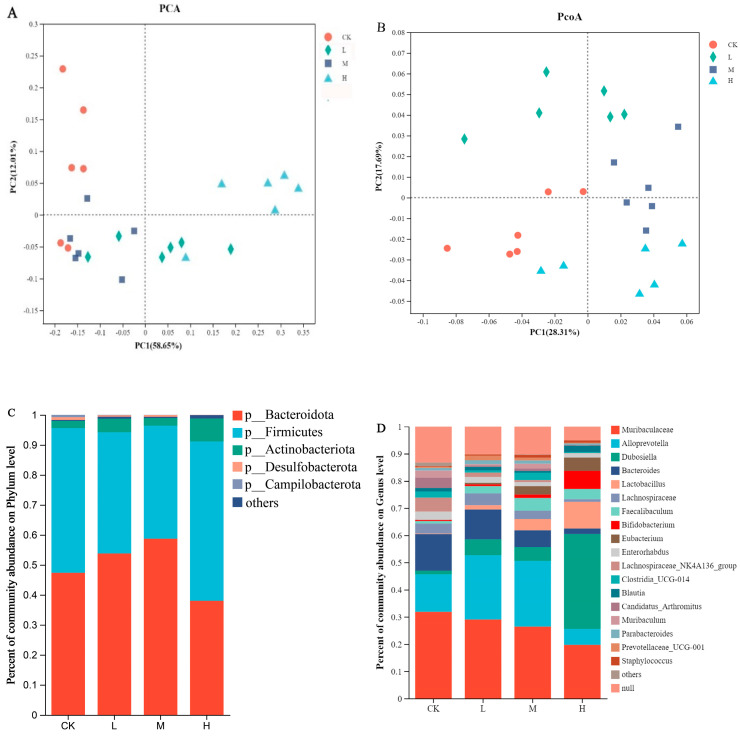

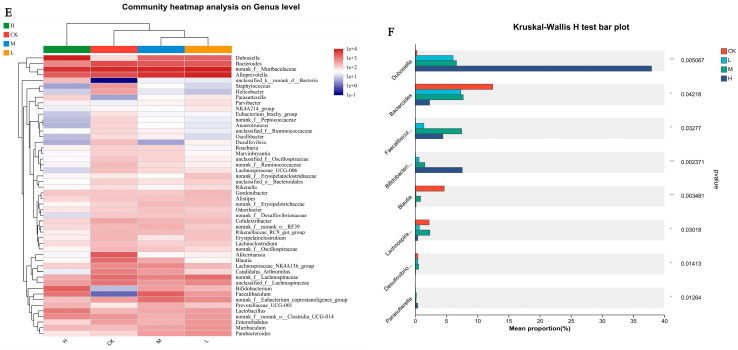
Effects of CSRPs on gut microbiota in mice. Control group was intragastrically administered with normal saline, L group was intragastrically administered with low-dose CSRP, M group was intragastrically administered with medium-dose CSRP, and H group was intragastrically administered with high-dose CSRP. (**A**) Principal component analysis (PCA); (**B**) calculating principal component analysis based on unweighted UNIFRAC distance (PCoA); (**C**) bacteria relative abundance at phylum level (%); (**D**) bacteria relative abundance at genus level (%); (**E**) bacterial clustering heat map; (**F**) significance test of difference between groups, * *p* < 0.05, ** *p* < 0.01.

Additionally, a principal component analysis (PCA) (Figure 4A) and the unweighted UNIFRAC distance were employed to calculate a Principal Coordinate Analysis (PCoA) (Figure 4B) to evaluate the community composition. The results demonstrated a significant separation between the groups, indicating a significant difference in gut microbiota with or without the intake of CSRPs. The analysis of the gut microbiota composition at the phylum level revealed that the dominant microbial phyla were Bacteroidota, Firmicutes, Actinobacteriota, Desulfobacterota, and Campilobacterota (Figure 4C). At the genus level, the dominant flora included *Muribaculaceae*, *Alloprevotella*, *Dubosiella*, *Bacteroides*, *Lactobacillus*, *Lachnospiraceae*, *Faecalibaculum*, *Bifidobacterium*, *Eubacterium*, and others. With increasing concentrations of CSRPs, the relative abundance of beneficial bacteria such as *Dubosiella*, *Lactobacillus*, *Faecalibaculum*, and *Bifidobacterium* increased, while the growth of *Bacteroides* and *Enterorhabdus* was inhibited (Figure 4D).

A cluster analysis revealed high composition similarity at the genus level within each group, with some genera showing high similarity and others displaying significant differences. As the concentrations of CSRPs increased, the differences between the groups became more pronounced (Figure 4E). A significant difference test analysis of gut microbiota at the genus level was conducted between the groups, and the results indicated significant differences in the bacterial composition of gut microbiota at the genus level in mice receiving different intragastric doses. This included *Dubosiella*, *Bacteroides*, *Faecalibaculum*, *Bifidobacterium*, *Trichospirillum, Eubacterium*, *Segmented filamentous bacteria*, *Faecalibacterium*, and *Enterorhabdus* (one-way ANOVA) (Figure 4F).

## 4. Discussion

Physicochemical changes in prebiotics can have a significant impact on the composition, digestive function, and immune response of the gastrointestinal microbiota. Prebiotics are selectively utilized by beneficial microorganisms in the host and have various health benefits. They can enhance resistance to pathogens, regulate immune function, improve mineral absorption, promote intestinal function, affect metabolism, and contribute to satiety [21]. Probiotics, on the other hand, play a role in immune regulation, the production of organic acids and antimicrobial compounds, interactions with resident microbiota, interactions with the host, improvement in intestinal barrier integrity, and enzyme formation [2]. Stellariae Radix is a traditional Chinese medicine, but its polysaccharides have been rarely studied. Our research has demonstrated that Stellariae Radix contains abundant water-soluble polysaccharides and possesses free radical scavenging effects [16]. In this study, we found that CSRPs can promote the growth of *Lactobacillus acidophilus* and *Bifidobacterium longum* in an MRS medium. This was evident from the significant increase in the sugar consumption and clump count of the two probiotics, as well as the reduction in pH in the sugar-free MRS medium. The PI values also indicated that the addition of 10% crude polysaccharides in the medium had the most pronounced effect on promoting the growth of probiotics. Therefore, our in vitro evaluation of prebiotic activity showed that CSRPs exhibited stronger prebiotic activity than inulin in promoting the growth of these two probiotics. This provides a theoretical basis for the potential development of Stellariae Radix as a prebiotic drug in the fields of food, healthcare products, and animal feed.

The gut microbiota plays a crucial role in human health and is associated with various diseases. The treatment and prevention of many intestinal-related diseases using probiotics and prebiotics involve modulating the microbiota and/or its functions, such as increasing the proportion of probiotics in the intestine and regulating the intestinal microenvironment [22]. In the intestine, Firmicutes and Bacteroidetes are the dominant bacterial phyla, and their proportion changes have been studied in relation to diseases such as obesity. Obesity is characterized by an increase in the proportion of Firmicutes and Bacteroidetes in the gut microbiota [23]. In our study, the results of the alpha diversity analysis showed that the low-dose and medium-dose groups increased the diversity and richness of the gut microbiota compared to the control group, while the high-dose group reduced them. At the phylum level, the low-dose and medium-dose groups reduced the abundance of Firmicutes and increased the abundance of Bacteroidetes, thereby reducing the Firmicutes/Bacteroidetes ratio. This suggests that low and medium doses of CSRPs in mice may have a preventive and therapeutic effect on obesity by modulating the intestinal environment. Firmicutes and Bacteroidetes represent both beneficial and potentially harmful bacteria, and their relative abundance lies between the optimal levels [24]. Therefore, the reduction in the diversity and richness of the gut microbiota observed in the high-dose group may be due to a decrease in the abundance or even the elimination of certain harmful bacteria.

In the analysis of bacterial composition at the genus level, the hypothesis was confirmed. With an increase in the concentration of crude polysaccharides, the relative abundance of *Dubosiella*, *Faecalibaculum, Bifidobacterium*, and *Lactobacillus* significantly increased, while harmful bacteria such as Enterorhabdus decreased significantly. It has been reported that *Dubosiella*, *Bifidobacterium*, and *Lactobacillus* are positively correlated with preventing and improving obesity and related diseases [25]. Zhu et al. found that *Dubosiella* can improve abnormal indexes in obese mice induced by a high-fat diet, reduce LDL-C, TG, and body weight, improve the digestion and absorption ability of glucose in mice, and reduce the expression levels of lipid metabolism genes such as CD36, FASN, and PPARγ in the liver, thereby alleviating the disorder of lipid metabolism caused by obesity [24]. Additionally, *Dubosiella* can increase the abundance of beneficial microorganisms (*Bifidobacterium* and *Lactobacillus*) in obese mice, improve the disorder of intestinal flora structure caused by a high-fat diet, improve intestinal metabolism and immunity, and reduce the occurrence of bacterial infections and other diseases. Numerous studies have also found that the content of *Dubosiella* increases during the treatment of obesity [26,27,28,29,30,31,32]. It has been proven to improve the metabolism of the body, normalize blood lipid metabolism, and increase the content of short-chain fatty acids (SCFAs) such as butyrate. Butyrate has the ability to promote the conversion of white adipose tissue (WAT) to brown adipose tissue (BAT). WAT stores energy, while BAT uses energy for heat production, resulting in increased energy consumption by the host and the degradation of fat by regulating the host’s TCA cycle. In this study, with the increase in CSRP concentration, the abundance of *Lactobacillus*, *Bifidobacterium*, and *Dubosiella* in the intestinal tract of the mice significantly increased. Therefore, this further supports the speculation that CSRPs can regulate the gut microbiota and potentially treat obesity.

Furthermore, certain intestinal microorganisms (*Bacteroides*, *Prevotella*, and *Lactobacillus*) play a crucial role in hydrolyzing polysaccharides into soluble cytokines [33]. The microbial fermentation of dietary carbohydrates, including prebiotics, promotes the production of SCFAs [34]. SCFAs are well-known signaling molecules. Firstly, SCFAs create an acidic environment in the colon, inhibiting the growth of pathogens. Secondly, SCFAs can regulate the growth and differentiation of intestinal cells and provide them with energy [35,36]. Studies have shown that *Bacteroides* can stimulate the production of propionic acid, which limits acetate synthesis and reduces SCFA synthesis, resulting in a negative correlation between SCFA content and the number of *Bacteroides* in the intestine [37]. In this study, compared to the control group, the treatment groups significantly reduced the relative abundance of *Bacteroides* in the intestine (*p* < 0.05) and inhibited its growth with increasing dosage. This indicates that crude polysaccharides can effectively inhibit the growth of Bacteroides, providing a theoretical basis for further studying the effects of SRPs on the SCFA content in the intestine.

## 5. Conclusions

In summary, this study provides a solid foundation for understanding the prebiotic effect of CSRPs. It demonstrates that CSRPs have the ability to enhance the growth of beneficial bacteria in mice, surpassing the prebiotic activity of inulin in vitro. Additionally, CSRPs have the potential to regulate the abundance and diversity of gut microbiota in mice. These findings suggest that a CSRP could be utilized as a prebiotic to modulate the gut microbiota, paving the way for the further exploration and utilization of Stellariae Radix as a valuable medicinal resource. This study provides a crucial theoretical basis for future research on CSRPs.

## 6. Patents

Peng, L., Wang, H., Song, L., Feng, L., Niu, P.L., Li, Z.K., Li, Y.Q., Li, H.S. & Wu, W., et al. A crude polysaccharide of Stellariae Radix with prebiotic activity, and its preparation method [P]. China: CN115677874A, 3 February 2023.

## Figures and Tables

**Table 1:** Nonlinear fitting parameters of growth kinetics equation.

.	Parameters	A_1_	A_2_	X_0_	p	R^2^
*Lactobacillus acidophilus*	2.5% inulin	0.024 ± 0.06	0.727 ± 0.02	4.892 ± 0.55	3.951 ± 1.23	0.967
	2.5% Glc	0.024 ± 0.06	1.140 ± 0.03	5.668 ± 0.46	2.857 ± 0.54	0.985
	2.5% CSRP	0.021 ± 0.03	0.954 ± 0.01	4.307 ± 0.16	4.035 ± 0.37	0.996
	5% CSRP	0.032 ± 0.04	1.391 ± 0.02	5.244 ± 0.20	4.138 ± 0.54	0.996
	10% CSRP	0.008 ± 0.02	1.872 ± 0.02	7.345 ± 0.12	3.792 ± 0.20	0.999
	15% CSRP	0.009 ± 0.02	1.801 ± 0.02	8.495 ± 0.17	3.930 ± 0.27	0.998
*Bifidobacterium longum*	2.5% inulin	0.044 ± 0.04	1.923 ± 0.13	25.043 ± 1.34	4.058 ± 0.66	0.991
	2.5% Glc	0.027 ± 0.04	1.487 ± 0.04	20.010 ± 0.42	4.181 ± 0.36	0.997
	2.5% CSRP	0.005 ± 0.04	2.192 ± 0.05	14.899 ± 0.31	6.441 ± 0.74	0.995
	5% CSRP	0.020 ± 0.03	2.217 ± 0.02	11.330 ± 0.14	7.986 ± 0.67	0.998
	10% CSRP	0.002 ± 0.05	2.360 ± 0.05	11.820 ± 0.26	6.017 ± 0.69	0.996
	15% CSRP	0.016 ± 0.03	1.711 ± 0.05	17.966 ± 0.35	7.601 ± 0.96	0.990

Note: parameters’ meanings were shown in experimental methods 2.3.

**Table 2:** Effects of CSRPs on the growth of Lactobacillus acidophilus and Bifidobacterium longum.

Bacterial Strain	Control Group (%)		Concentration of CSRP (%)			
	2.5% Inulin	2.5% Glc	2.5%	5%	10%	15%
*Lactobacillus acidophilus*	1.36 ± 0.10 b	0.95 ± 0.05	0.8 ± 0.17 a	1.76 ± 0.02 c	2.39 ± 0.03 e	2.06 ± 0.02 d
*Bifidobacterium longum*	1.49 ± 0.20 a	0.84 ± 0.03	2.08 ± 0.03 b	2.36 ± 0. 03 d	2.44 ± 0.02 e	2.27 ± 0.03 c

Note: values are expressed as mean values of log10 ± SD (CFU) per milliliter of MRS. And because a, b, c, d, and e are in the same row, the average values of different letters were significantly different when *p* < 0.05, *n* = 3.

**Table 3:** Effects of CSRPs on PI values of probiotics.

	2.5% CSRP	5% CSRP	10% CSRP	15% CSRP
*Lactobacillus acidophilus*	0.85 ± 0.02 c	1.28 ± 0.01 b	1.73 ± 0.01 a	1.72 ± 0.01 a
*Bifidobacterium longum*	2.16 ± 0.03 b	2.16 ± 0.01 b	2.34 ± 0.02 a	1.62 ± 0.07 c

Note: values are Probiotic value ± SD of CSRP, because a, b, and c are in the same row, the average values of different letters were significantly different when *p* < 0.05, *n* = 3.

**Table 4:** α-diversity index of gut microbiota in each group.

Sample	Richness		Diversity		Coverage
	Chao1	Ace	Shannon	Simpson	
CK	288.26 ± 26.66	286.53 ± 27.36	3.40 ± 0.39	0.10 ± 0.04	0.999
L	312.61 ± 19.09	306.36 ± 21.03	3.38 ± 0.36	0.08 ± 0.02	0.999
M	316.24 ± 38.84	308.36 ± 39.18	3.38 ± 0.81	0.06 ± 0.36	0.999
H	284.81 ± 17.29	284.57 ± 17.29	2.84 ± 0.28	0.18 ± 0.05	0.998

Data are presented as mean ± SD (*n* = 6) and compared with control group.

## Data Availability

The original contributions presented in the study are included in the article, and further inquiries can be directed to the corresponding authors.
